# Impact of multilingualism on cardiac autonomic activity, working memory and trait anxiety: a pilot study

**DOI:** 10.1007/s10339-026-01392-y

**Published:** 2026-10-01

**Authors:** Tonantzin Itzel Cabrera-Ontiveros, Diana Estefanía García-Cárdenas, Claudia Ivette Ledesma-Ramírez, Eric Alonso Abarca-Castro, Ana Karen Talavera-Peña, José Javier Reyes-Lagos

**Affiliations:** 1 Departamento de Ciencias de la Salud, División de Ciencias Biológicas y de la Salud, Universidad Autónoma Metropolitana (UAM-L), Lerma de Villada, Mexico; 2 Facultad de Medicina, Universidad Autónoma del Estado de México (UAEMéx), Toluca de Lerdo, Mexico; 3 Sección de Bioelectrónica, Departamento de Ingeniería Eléctrica, Centro de Investigación y de Estudios Avanzados del Instituto Politécnico Nacional (Cinvestav Unidad Zacatenco), Mexico City, Mexico

**Keywords:** Multilingualism, Heart rate variability, Working memory, Anxiety

## Abstract

Recent years have witnessed a growing interest in how multilingualism modifies executive functions. Heart rate variability (HRV), a noninvasive and innocuous indicator of cardiac autonomic function, can also capture individual differences that link cognitive performance with physiological and emotional profiles. This pilot study aimed to analyze the association of multilingualism with cognitive, autonomic, and emotional regulation during working memory (WM) tasks, while also considering trait anxiety (Trait-A). Nineteen young adults were classified as a monolingual group (MON, *N* = 9) or a multilingual group (MUL, *N* = 10). Participants completed the State‑Trait Anxiety Inventory (STAI) and the Neuropsychological Battery of Executive Functions and Frontal Lobes (BANFE‑3). They then performed an N-back task at three difficulty levels while HRV linear and nonlinear indices were computed. MON exhibited high Trait‑A, whereas MUL showed moderate Trait‑A. No significant differences were observed in the BANFE-3 subtests, but MUL achieved greater accuracy at the 2-back level of the N-back task. HRV indices showed a load-dependent decline in both groups; within-group analyses, however, revealed that MON and MUL participants showed different within-group autonomic response patterns to cope with the rising cognitive load. These findings suggest that sustained multilingual experience may be associated with differences in autonomic–cognitive responses and attenuated anxiety-related disruptions, underscoring the value of HRV in characterizing individual variability in physiological and behavioral responses.

**Supplementary Information:**

The online version contains supplementary material available at https://doi.org/10.1007/s10339-026-01392-y.

## Introduction

Language experience is a lifelong factor that reshapes brain structure (Bialystok 2017), mainly in terms of executive functions (Kovács and Mehler 2009; Morales et al. 2013; Comishen and Bialystok 2021; Fyndanis et al. 2023). Thus, compared with monolinguals (MON), bilinguals continuously engage language-control processes, which may contribute to differences in executive functioning (Kroll et al. 2012; Green and Abutalebi 2013; Bialystok 2017) primarily supported by prefrontal-cortex networks (Fuster 2002). The existing literature has mainly studied them using three primary constructs: cognitive flexibility or set shifting, response inhibition, and working memory (WM) (Miyake et al. 2000). WM allows information to be temporarily maintained and stored for later processing and manipulation when performing complex cognitive tasks (Baddeley 2003).

In this context, various studies have examined whether the dominance of more than one language confers an advantage on WM. Previous research has demonstrated that being bilingual or multilingual (MUL) improves performance in WM tasks (Bialystok et al. 2004; Morales et al. 2013; Comishen and Bialystok 2021; Fyndanis et al. 2023), mainly in the visuospatial component (Cockroft et al. 2019), but this advantage seems to disappear in verbal tasks (Bialystok et al. 2008; Calvo et al. 2016), while other studies have found no differences (Namazi and Thordardottir 2010; Engel de Abreu 2011; Yang 2017). Thus, the putative “bilingual advantage” in WM remains contentious.

One of the most widely used experimental paradigms for assessing WM is the N-back task, which can modulate cognitive load by increasing the number of elements to retain and has been shown to activate prefrontal brain regions associated with WM (Yeung and Han 2023). Participants must continually update and reallocate resources to manipulate and maintain attention on target information, processes inherent to WM (Daffner et al. 2011). Therefore, the N-back task is an appropriate paradigm for evaluating the increase in WM cognitive load between MON and MUL participants. This study also employs the Neuropsychological Battery of Executive Functions and Frontal Lobes, 3rd edition (BANFE-3), to measure additional components of WM and to provide a broader perspective on this executive function in relation to multilingualism.

Better performance on executive tasks has been associated with physiological and emotional self-regulation processes. In this respect, the Neurovisceral Integration Model (NIM) provides a perspective that relates cognitive performance with autonomic and emotional regulation (Thayer and Lane 2000; Thayer et al. 2009). Although multilingualism has been studied mainly in relation to executive functions, relatively less attention has been given to its simultaneous examination from cognitive, emotional, and autonomic perspectives. This study aims to contribute to this area by examining multilingualism across these three domains. 

The NIM posits that heart rate variability (HRV) may serve as an index of neurovisceral integration and has been associated with prefrontal regulation of cognitive and affective processes. HRV is defined as the time variability between adjacent heartbeats and reflects the autonomic modulation of cardiac activity (Shaffer and Ginsberg 2017). It is also an indicator of a healthy functional state, because optimal levels reflect the body’s capacity for self-regulation and adaptability (McCraty and Shaffer 2015). In relation to the NIM, it has been found that greater resting vagally mediated HRV has been linked to enhanced cognitive performance on WM tasks (Thayer et al. 2009) and that an effortful attentional control, as well as increased mental workload, produces a decrease in HRV (Porges and Raskin 1969; Hansen et al. 2003; Wang et al. 2005; Byrd et al. 2014; Luque-Casado et al. 2016). These changes reflect the body’s ability to adapt to the demands of the task.

Within the connection between the prefrontal cortex and emotional regulation, anxiety is of particular interest in this study because its intersection with multilingualism remains understudied in executive-function research.

Regarding cognitive functioning, anxiety can have implications in decision making, decreased attentional control, and WM (Clark and Beck 2012). Specifically, it has been found that individuals with higher levels of anxiety, whether trait (Trait-A) or state (State-A), tend to make more mistakes in WM tasks and those requiring greater attention (Moran 2016; Lukasik et al. 2019). Studies have suggested that anxiety interferes with cognitive processes primarily by decreasing the ability to prevent irrelevant information from accessing WM and attentional resources that should be allocated to tasks (Eysenck et al. 2007; Qi et al. 2014; Moran 2016). Also, an association between anxiety and HRV has been reported, with lower HRV being observed in individuals with anxiety disorders (Chalmers et al. 2014). Within the neurovisceral integration framework, reduced HRV has been associated with lower cognitive flexibility in allocating attention and responding to environmental demands (Thayer and Lane 2000).

Previous research has mainly focused on multilingualism in relation to executive functions (Morales et al. 2013; Fyndanis et al. 2023) and neuroanatomical correlates (Wong et al. 2016), without considering autonomic factors such as HRV, which are known to influence WM performance (Zeng et al. 2023), or emotional variables such as anxiety, which impact executive functioning (Moran 2016; Warren et al. 2021).

This gap hampers our understanding of the autonomic and emotional mechanisms that may underlie the so-called “bilingual advantage”. Our experimental design analyzes and compares MON and MUL young adults across HRV indices, WM performance, and anxiety (Trait-A and State-A). Furthermore, in contrast to prior studies that rely on a single measure of WM, the present study employs the BANFE-3 in addition to the N-back tasks and simultaneous HRV assessment, enabling evaluation of physiological responses to increasing cognitive load. We expect that MUL participants, compared with MON participants, will demonstrate (i) better overall WM performance, (ii) more adaptive HRV responses during cognitive effort, and (iii) lower levels of Trait-A. Integrating these three domains may provide a more comprehensive characterization of multilingualism than cognitive performance alone.

## Methodology

### Study design

**Fig. 1:**
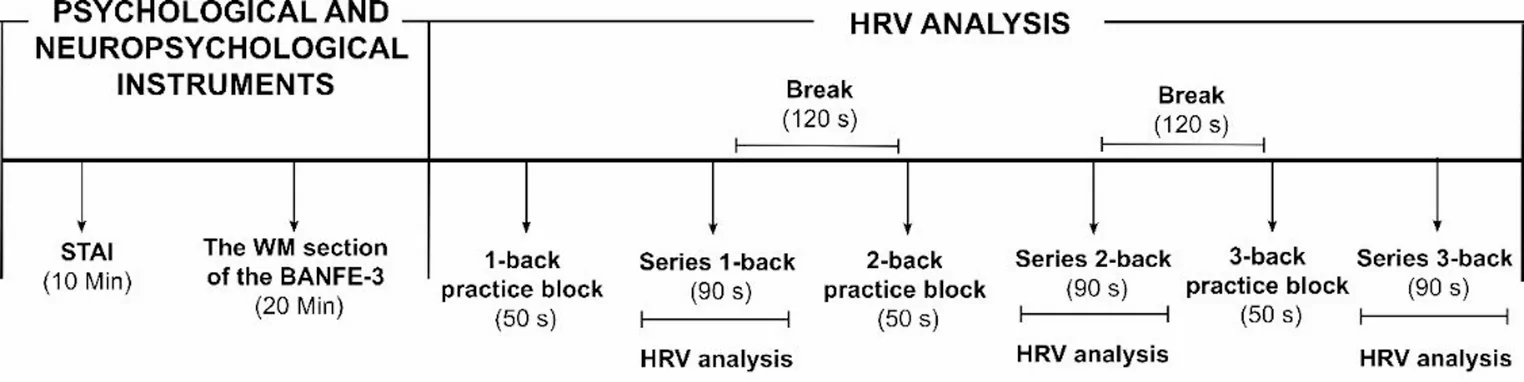
Timeline of the study protocol. MON and MUL participants completed psychological and neuropsychological assessments (STAI and the WM section of the BANFE-3), followed by the N-back task at three difficulty levels. HRV analysis was conducted during each series block. Breaks were included between tasks to allow for autonomic recovery. For simplicity, one of the two 90-s series performed at each N-back level is shown

The study design used was exploratory, cross-sectional, and comparative. This research was conducted from March to October 2024. MON and MUL participants were recruited via a flyer distributed on social media, which encouraged them to complete an online form to provide personal details and indicate the number of languages they spoke. Those meeting the inclusion criteria were invited to an online interview, where individual and sociodemographic information, foreign language skills, and medical history were collected. Afterwards, a face-to-face session was scheduled for each participant, lasting about two hours. The protocol’s methodological pipeline is illustrated in Fig. 1. All procedures were conducted at the facilities of the School of Medicine, Universidad Autónoma del Estado de México (UAEMéx), in Toluca, Mexico. The study protocol was approved by the Research Ethics Committee of the School of Medicine, Universidad Autónoma del Estado de México (registration no. 015.2023). All participants provided written informed consent before participation.

Initially, all the participants completed the following psychological and neuropsychological assessments: the State-Trait Anxiety Inventory (STAI) and the WM section of the BANFE-3.

Lastly, an N-back task was performed at three difficulty levels, with simultaneous electrocardiogram (ECG) recordings used to assess autonomic nervous system activity via HRV. Ultra-short-term HRV was analyzed for each 90-second series, excluding practice periods. Short breaks of 120 s were included between blocks to allow for autonomic recovery and reduce carry-over effects.

### Participants

Inclusion criteria required participants to (i) be Mexican nationals and current residents of the Toluca Valley or Mexico City; (ii) be between 18 and 35 years of age; (iii) have completed at least a high school education; (iv) for the MON group, report Spanish as their native language; (v) for the MUL group, provide certification validating proficiency in a second or third language; and (vi) complete all administered questionnaires. Exclusion criteria included (i) poor-quality ECG recordings; (ii) native English speakers; (iii) self-reported chronic, hypertensive, or neurological disorders; (iv) current use of medications that affect the central nervous system; and (v) consumption of alcohol, coffee, tobacco, or drugs within 24 h before testing. Initially, 26 participants were recruited; however, 7 were excluded: 4 due to inadequate ECG signal quality, 2 taking psychotropic medication, and 1 with incomplete data recorded during the N-back task, leaving a final sample of 19 participants. These 19 Mexican, non-native English speakers were classified into the MON (*N* = 9) or MUL (*N* = 10) groups. MUL participants held certification showing at least B2 English proficiency according to the Common European Framework of Reference for Languages (CEFR), and, where applicable, at least B1 proficiency in another language. Although MON participants had been exposed to English in elementary or high school, their proficiency remained at the basic A1 level, thereby justifying their classification as MON.

### Psychological and neuropsychological instruments

#### State-trait anxiety inventory (STAI)

The STAI comprises two scales that assess distinct aspects of anxiety: Trait-A, which reflects an individual’s typical emotional state, and State-A, which captures their emotional state at a specific moment (Spielberger et al. 1970). This study utilized the Spanish version, which has been validated for the Mexican population and has demonstrated high internal consistency. Cronbach’s alpha coefficients range from 0.83 to 0.92 for the State-A scale and from 0.86 to 0.92 for the Trait-A scale (Spielberger and Díaz-Guerrero 1975). Each scale consists of 20 items rated on a 4-point Likert-type scale. The response options were: “Not at all,” “Somewhat,” “Quite a bit,” and “Very much”. Scores on the scale range from 20 to 80. A score below 30 indicates low anxiety, scores between 30 and 44 indicate moderate anxiety, and scores of 45 or above indicate high anxiety (Castillo Pimienta et al. 2016).

#### Neuropsychological battery of executive functions and frontal lobes 3rd edition (BANFE-3)

WM was evaluated with the WM section of the BANFE-3, created by Flores et al. (2020). This section includes various tasks to assess different aspects of WM, such as the Self-Directed Pointing Task, Alphabetical Ordering of Words, Consecutive Subtraction/Addition, and the Visuospatial Working Memory Test. The BANFE-3 has a convergent and clinical validity coefficient of 0.80.

The Self-Directed Pointing task assesses visuospatial WM capacity through a self-initiated activity. The Alphabetical Ordering of Words test measures the ability to retain information in WM and manipulate it mentally. The Consecutive Subtraction/Addition test evaluates the capacity to perform sequential and inverse mental operations. Finally, the Visuospatial Working Memory test examines the ability to remember the identity and spatial arrangement of objects in a specific context order (Flores et al. 2020).

### Acquisition of ECG and ultra-short analysis of HRV

For HRV analysis, ECGs were recorded using a g.USBamp biosignal amplifier (g.tec medical engineering GmbH, Austria). Only the lead DI, derived from Einthoven’s triangle, was acquired (Einthoven et al. 1950). The ECG signal was sampled at 512 Hz, and filtered using an 8th-order Butterworth bandpass filter (0.1–100 Hz), together with a 4th-order Chebyshev notch filter (58–62 Hz) to attenuate 60-Hz power-line interference (Huhta and Webster 1973; Chu et al. 2023). Signal acquisition and recording were managed using the BCI2000 software system, version v3.6.beta.R7385 (National Center for Adaptive Neurotechnologies, Albany, NY, USA).

Kubios HRV analysis software version 2.2 (University of Kuopio, Kuopio, Finland) was used to extract the RR-interval time series from the automatically detected R-waves in the ECG. This was achieved through its built-in QRS detection algorithm, which is based on the Pan–Tompkins method (Pan and Tompkins 1985).

The “very low threshold” artifact correction algorithm was applied to identify and correct missing beats, using default RR interval interpolation at 4 Hz and a 16th-order autoregressive (AR) spectral model without factorization for the spectral analysis. This method was used because it yields a higher-resolution spectrum from short data records, as in this study (Miranda Dantas et al. 2012). The HRV indices analyzed included both linear and nonlinear measures: mean RR, standard deviation of all RR intervals (SDNN), mean heart rate (HR), square root of the mean squared differences between successive RR intervals (RMSSD), the proportion of NN50 relative to the total number of RR intervals (pNN50), RR triangular index (i.e., the integral of the density distribution divided by its height), and triangular interpolation of NN intervals (TINN). Frequency-domain indices included power in the low-frequency (LF; 0.04–0.15 Hz) and high-frequency (HF; 0.15–0.40 Hz) bands. These conventional band limits are widely used for short-term human HRV analysis and facilitate comparability across studies by separating oscillatory components typically observed in these frequency ranges (Tarvainen et al. 2014). Nonlinear indices included the Poincaré plot indices that measure the standard deviation of short (SD1) and long-term (SD2) HRV.

Time-domain, frequency-domain, and nonlinear indices were selected due to their widespread use in the literature and because most of them are recommended for fundamental linear and nonlinear HRV analysis (Shaffer and Ginsberg 2017). HRV indices were computed separately for each 90-s N-back series to time-lock autonomic estimates to the cognitive-load condition and to avoid mixing across blocks, and then averaged within each difficulty level for statistical analysis. While traditional short-term HRV guidelines usually rely on approximately 5-minute recordings, evidence indicates that ultra-short segments of about 1–2 min can still yield reliable results for mean HR, mean RR, and vagally mediated time-domain and Poincaré metrics like RMSSD and SD1. Therefore, this study mainly focused on these indices when analyzing ultra-short HRV data, while frequency-domain measures were explored more tentatively due to the shorter window length (Munoz et al. 2015).

### N-back task

The N-back task was presented using BCI2000 version v3.6.beta.R7385, at three difficulty levels (1-, 2-, and 3-back), while ECG signals were recorded simultaneously. Before starting the task, participants received a general explanation, and before each level, an instructional video specific to that condition was shown.

Participants were seated approximately 50 cm from the computer screen, on which stimuli were displayed centrally (1600 × 900 pixels) and occupied 50% of the screen height. Digits from 1 to 9 appeared in white on a black background for 500 ms, followed by a 1500 ms interstimulus interval showing only the black background. During the task, participants used a left mouse click to indicate whether the current digit was a target—that is, whether it matched a previously presented stimulus. In the 1-back condition, the target was the immediately preceding digit; in the 2-back and 3-back conditions, it was the digit shown two or three trials earlier, respectively.

**Fig. 2:**
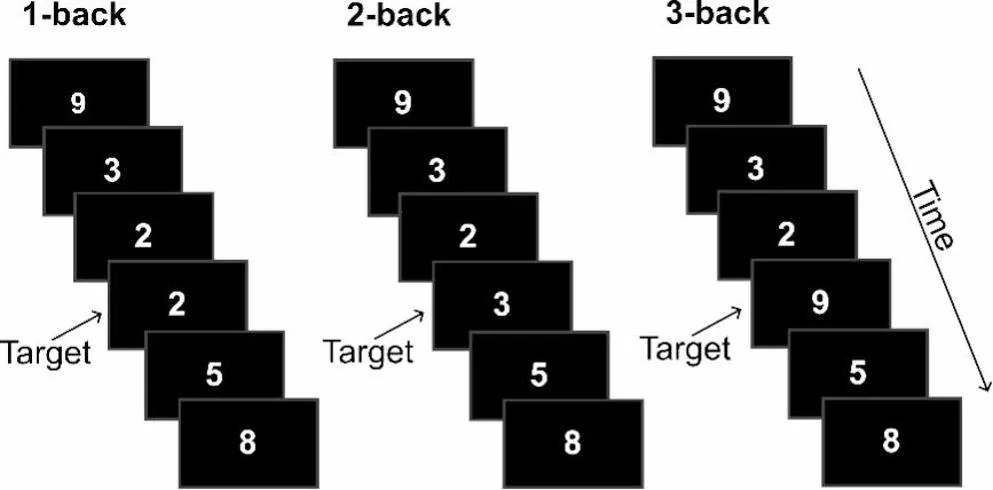
Representative example of stimulus presentation and task conditions for the N-back task. Each panel illustrates the structure of the 1-back, 2-back, and 3-back levels, where participants were instructed to identify a target digit that matched the one presented one, two, or three trials earlier, respectively. Stimuli were presented sequentially over time

Each level consisted of two 90-second series comprising 45 trials with 15 targets. For each difficulty level, performance measures from the two series were averaged before statistical analysis. To confirm task comprehension, a 50-second practice block (24 trials, 8 targets) was administered before the main series. This practice was repeated as needed until the participant understood the instructions. All participants completed the same practice block, whereas the main series was individualized. For this purpose, random 45-trial sequences were generated in MATLAB version R2023a (The MathWorks Inc., Natick, Massachusetts), creating a pool of 15 unique series per participant for each level, from which two series were selected for each participant (Fig. 2).

### Performance metrics across N-back task levels

N-back performance was quantified at all three difficulty levels (1-, 2-, and 3-back) using the percentage of true positives (TP), percentage of false positives (FP), percentage of correct responses (C), and response time (RT), calculated as follows:

1 $$\:\%\:TP=\frac{TP}{FN+TP}\cdot\:100$$

In Eq. (1), FN denotes false negatives. The sum of TP and FN was 15, as it was the number of targets set for each series.

2 $$\:\%\:FP=\frac{FP}{TN+FP}\cdot\:100$$

In Eq. (2), TN denotes true negatives. The sum of FP and TN was 30, as it was the number of stimuli that were not targets.

3 $$\:\%\:C=\frac{TP+TN}{45}\cdot\:100$$

In Eq. (3), % C represents the percentage of correct responses or overall accuracy, calculated based on the sum of correct identifications (TP + TN) over the total number of trials (45).

### Statistical analysis

The normality of each variable was assessed using the Anderson–Darling test. Scores from the psychological questionnaire, the neuropsychological WM subtests of the BANFE-3, and the complete set of HRV indices were compared between the MON and MUL groups. Variables that followed a normal distribution and met the assumption of homogeneity of variances, as determined by Levene’s test, were analyzed using independent samples *t*-tests; otherwise, the Mann–Whitney *U* test was applied.

To examine the effect of cognitive load on autonomic activity within each group (repeated measures), HRV indices were compared across the three N-back levels (1-, 2-, and 3-back). For variables with normally distributed data, a one-way repeated measures analysis of variance (ANOVA) was performed. Greenhouse–Geisser corrections were applied when the assumption of sphericity was violated, and significant main effects were followed by Fisher’s least significant difference (LSD) post hoc comparisons. Variables failing to meet normality assumptions were analyzed with Friedman’s test. When a significant omnibus result emerged, uncorrected Dunn’s tests identified specific differences. Significance was defined at α = 0.05 (two-tailed).

Effect sizes were reported as Hedges’ g for parametric between-group comparisons and Cliff’s δ for non-parametric between-group comparisons. For within-group (paired) post hoc contrasts, repeated-measures Hedges’ g (g_rm) and/or rank-biserial correlation (r_rb) were reported as appropriate.

Given the exploratory nature of this pilot study and the limited sample size (*n* = 19), post hoc pairwise comparisons were conducted without formal correction for multiple testing (Fisher’s LSD and uncorrected Dunn’s tests), following significant omnibus effects. This decision was made to reduce the risk of type II error in this hypothesis-generating study (Rothman 1990; Li et al. 2016).

Furthermore, given the exploratory nature of the study, sample size was not determined by a priori power estimation (Leon et al. 2011). Post hoc power analyses were conducted to assess the statistical sensitivity of the final sample. As discussed in the literature, post hoc power analyses are frequently requested when nonsignificant results raise concerns about potential false-negative findings due to limited sample size (Heckman et al. 2022). Accordingly, the post hoc power analysis is reported here solely to address this concern and should be interpreted with caution, without being used to support inferential conclusions.

All statistical analyses were conducted using MATLAB R2023a (The MathWorks Inc., Natick, MA, USA).

## Results

Nineteen participants fulfilled all inclusion criteria and produced analyzable data. The MON group had a mean age of 22 ± 4 years and included six women and three men, whereas the MUL group had a mean age of 22 ± 3 years and comprised six women and four men. Additional sociodemographic and health-related information is summarized in Table 1. MUL exhibited a more diverse linguistic profile, a higher proportion of postgraduate education, a greater prevalence of family histories of diabetes and hypertension, and a markedly higher frequency of regular physical activity (90% vs. 44% in MON).

In addition, Table 2 summarizes factors related to multilingual language experience. 60% of MUL participants had advanced English proficiency (C1 or C2), whereas 40% had upper-intermediate proficiency (B2). Proficiency in the second and third foreign languages ranged from B1 to C1. The mean age of acquisition was 6.7 ± 4.47 years for English, 14.75 ± 9.47 years for the second foreign language, and 17.67 ± 7.77 years for the third foreign language. Mean frequency of use was 6.6 ± 0.84, 1.75 ± 0.96, and 4.33 ± 3.06 days per week, respectively.

**Table 1:** Sociodemographic and Health-Related Characteristics of the MON and MUL Participants

Variable	Group	
	MON (*N* = 9)	MUL (*N* = 10)
*Number of foreign languages known*		
One language (English)	N/A	6 (60%)
Two languages	N/A	1 (10%)
Three or more languages	N/A	3 (30%)
*Level of education*		
University	9 (100%)	9 (90%)
Master’s degree	N/A	1 (10%)
*Family medical history of chronic degenerative diseases*		
Diabetes	5 (55%)	8 (80%)
Hypertension	3 (33%)	8 (80%)
Cancer	2 (22%)	4 (40%)
Regular physical activity	4 (44%)	9 (90%)

Data are presented as N (%) for categorical variables. MON = monolingual; MUL = multilingual; N/A = not applicable. Regular physical activity was defined as engaging in at least 150 min of moderate-intensity exercise per week

**Table 2:** Factors related to multilingual language experience

Variable	Number of foreign languages known		
	English *N* = 10	Second foreign language *N* = 4	Third foreign language *N* = 3
*Language proficiency*			
B1	N/A	1 (25%)	1 (33.33%)
B2	4 (40%)	1 (25%)	1 (33.33%)
C1	3 (30%)	2 (50%)	1 (33.33%)
C2	3 (30%)	N/A	N/A
Age of acquisition (years)	6.7 ± 4.47	14.75 ± 9.47	17.67 ± 7.77
Frequency of use was self-reported (days per week)	6.6 ± 0.84	1.75 ± 0.96	4.33 ± 3.06

Data are presented as N (%) for categorical variables (Language proficiency) and as (M ± SD) for age of acquisition and frequency of use variables. N/A = not applicable

### Comparison of STAI scores between MON and MUL groups

Trait-A differed significantly between groups: MUL participants scored, on average, 18 points lower than their MON counterparts (*p* = 0.0011; δ = 0.8333), indicating a significantly lower dispositional tendency toward anxiety in MUL. Specifically, MUL participants reported an average score consistent with moderate anxiety (34.80 ± 8.23), while MON averaged a score indicative of high anxiety (53.00 ± 9.29).

State-A followed the same directional trend but did not reach statistical significance (36.89 ± 7.85 for MON vs. 31.00 ± 8.12 for MUL; *p* = 0.1274; g = 0.7035). The complete descriptive statistics are provided in Table 3.

### Comparison of BANFE-3 WM performance between MON and MUL groups

The total WM score from the BANFE-3 did not differ significantly between groups (104.22 ± 10.66 vs. 110.20 ± 7.81; *p* = 0.2661; δ = −0.3111). Similarly, no significant group differences were observed in the Self-Directed Pointing, Consecutive Subtraction/Addition, Visuospatial Working Memory, and Alphabetical Ordering of Words subtests (*p* ≥ 0.0681 for all). Full descriptive statistics are presented in Table 3.

**Table 3:** Anxiety (STAI) and BANFE-3 Measures in MON and MUL Groups

Variable	Group		*p*-value (between groups)	Effect size (between groups)
	MON *N* = 9 (M ± SD)	MUL *N* = 10 (M ± SD)		
*STAI*				
Trait-A (points)	53.00 ± 9.29	34.80 ± 8.23	0.0011**	δ = 0.8333
State-A (points)	36.89 ± 7.85	31.00 ± 8.12	0.1274	g = 0.7035
BANFE-3				
Total Score (points)	104.22 ± 10.66	110.20 ± 7.81	0.2661	δ = −0.3111
Self-directed pointing (points)	30.33 ± 3.35	29.80 ± 3.22	0.7283	g = 0.1550
Consecutive subtraction/addition (points)	53.00 ± 8.56	57.60 ± 6.54	0.1040	δ = −0.4444
Visuospatial working memory (points)	11.44 ± 1.88	11.20 ± 1.69	0.6119	δ = 0.1444
Alphabetical ordering of words (points)	9.44 ± 2.92	11.60 ± 1.84	0.0681	g = −0.8549

*MON* monolingual; *MUL* multilingual; *N* number of individuals; *STAI* State-Trait Anxiety Inventory; *Trait-A* trait anxiety; State-A State Anxiety; BANFE−3 Neuropsychological Battery of Executive Functions and Frontal Lobes, 3rd edition. *Significant at p < 0.05; ** p < 0.01

### Comparison of HRV between MON and MUL groups

The results of comparisons of HRV indices between groups are presented in Table 4.

Across all N-back levels, MON participants exhibited longer cardiac cycles than their MUL counterparts. For the 1-back condition, the mean RR interval was 804.81 ± 107.94 ms in MON vs. 735.58 ± 89.20 ms in MUL (*p* = 0.1444; g = 0.6716); similar trends were observed in the 2-back (786.95 ± 99.78 vs. 717.30 ± 77.69 ms; *p* = 0.1059; g = 0.7495) and 3-back blocks (780.26 ± 102.97 vs. 724.46 ± 83.92 ms; *p* = 0.2107; g = 0.5709). Correspondingly, mean HR tended to be lower in MON at 1-back (76.07 ± 9.83 vs. 82.94 ± 9.72 bpm; *p* = 0.1448; g = −0.6707), 2-back (77.62 ± 9.56 vs. 84.77 ± 8.97 bpm; *p* = 0.1107; g = −0.7384), and 3-back (78.32 ± 9.99 vs. 84.09 ± 9.95 bpm; *p* = 0.2250; g = −0.5526).

All other time-domain, frequency-domain, and non-linear HRV indices (SDNN, RMSSD, RR-triangular index, TINN, LF, HF, SD1 and SD2) showed no statistically significant group differences at any cognitive load (all *p* ≥ 0.05).

### Within-group comparison of HRV indices across cognitive load levels

The indices indicating statistically significant within-group differences are reported in Table 4.

In the MON group, cardiac cycle length decreased progressively with increasing cognitive demand. Mean RR intervals declined from 804.81 ± 107.94 ms in the 1-back block to 786.95 ± 99.78 ms in the 2-back († *p* = 0.0336) and further to 780.26 ± 102.97 ms in the 3-back (‡ *p* = 0.0077), yielding a significant overall effect (*p* = 0.0083). A corresponding increase in mean HR confirmed this chronotropic response (76.07 ± 9.83, 77.62 ± 9.56 †, and 78.32 ± 9.99 bpm ‡, *p* = 0.0077).

Among frequency-domain and non-linear indices, LF decreased from 470.56 ± 390.95 at 1-back to 369.61 ± 318.63 at 3-back (‡, omnibus *p* = 0.0446), while HF decreased from 1804.89 ± 3520.10 to 1256.06 ± 2900.47 over the same conditions (‡, omnibus *p* = 0.0319). None of the remaining HRV indices (SDNN, RMSSD, RR-triangular index, TINN, SD1, SD2) showed significant changes across blocks (all omnibus *p* ≥ 0.1690).

In the MUL group, mean RR and mean HR remained stable across the three task levels (*p* = 0.1806 and *p* = 0.2443, respectively). However, variability measures were sensitive to cognitive load. SDNN decreased from 41.86 ± 19.66 ms in 1-back to 34.61 ± 21.64 ms in 3-back (‡, omnibus *p* = 0.0367), and RMSSD declined from 34.31 ± 22.78 to 27.82 ± 17.00 ms (‡ *p* = 0.0253). A similar reduction was observed in the RR-triangular index (9.56 ± 2.66, 7.81 ± 2.35†, 7.59 ± 3.33‡; *p* = 0.0032) and in TINN, which narrowed from 181.50 ± 73.37 ms at 1-back to 143.00 ± 74.09 ms at 3-back (‡, *omnibus* *p* = 0.0103; pairwise *p* = 0.0025).

Non-linear dynamics followed a comparable trend. SD1 decreased from 24.37 ± 16.19 at 1-back to 19.77 ± 12.08 at 3-back (‡ *p* = 0.0253), and SD2 declined from 53.37 ± 23.32 to 44.14 ± 27.84 (‡ *p* = 0.0254).

**Table 4:** Descriptive Statistics and Between-Group and Within-Group Comparisons of HRV Indices

Variable		Group		*p*-value (between groups)	Effect size (between groups)
		MON *N* = 9 (M ± SD)	MUL *N* = 10 (M ± SD)		
*HRV indices*					
Mean RR (ms)	1-back	804.81 ± 107.94	735.58 ± 89.20	0.1444	g = 0.6716
	2-back	786.95 ± 99.78^†^	717.30 ± 77.69	0.1059	g = 0.7495
	3-back	780.26 ± 102.97^‡^	724.46 ± 83.92	0.2107	g = 0.5709
SDNN (ms)	1-back	46.28 ± 36.56	41.86 ± 19.66	0.4470	δ = −0.2222
	2-back	44.12 ± 30.15	36.73 ± 19.28	0.7340	δ = 0.1000
	3-back	39.59 ± 30.57	34.61 ± 21.64^‡^	0.9682	δ = 0.0222
Mean HR (bpm)	1-back	76.07 ± 9.83	82.94 ± 9.72	0.1448	g = −0.6707
	2-back	77.62 ± 9.56^†^	84.77 ± 8.97	0.1107	g = −0.7384
	3-back	78.32 ± 9.99^‡^	84.09 ± 9.95	0.2250	g = −0.5526
RMSSD (ms)	1-back	53.38 ± 61.19	34.31 ± 22.78	0.9682	δ = −0.0222
	2-back	46.97 ± 49.87	28.18 ± 14.44	0.8421	δ = 0.0667
	3-back	43.89 ± 47.75	27.82 ± 17.00^‡^	0.9048	δ = 0.0444
RR triangular index	1-back	8.48 ± 3.21	9.56 ± 2.66	0.4326	g = −0.3528
	2-back	8.61 ± 3.09	7.81 ± 2.35^†^	0.5284	g = 0.2825
	3-back	8.17 ± 3.95	7.59 ± 3.33^‡^	0.7306	g = 0.1536
TINN (ms)	1-back	167.78 ± 102.07	181.50 ± 73.37	0.2502	δ = −0.3222
	2-back	170.00 ± 95.05	152.25 ± 70.75	0.6454	δ = 0.1333
	3-back	158.61 ± 109.25	143.00 ± 74.09^‡^	0.7174	g = 0.1615
LF (ms^2^)	1-back	470.56 ± 390.95	708.85 ± 464.78	0.2428	δ = −0.3333
	2-back	416.56 ± 312.49	529.80 ± 682.76	0.9048	δ = 0.0444
	3-back	369.61 ± 318.63^‡^	392.95 ± 306.93	0.8728	g = −0.0713
HF (ms^2^)	1-back	1804.89 ± 3520.10	618.25 ± 889.87	0.7197	δ = −0.1111
	2-back	1214.44 ± 2664.15	337.10 ± 334.89	0.6038	δ = −0.1556
	3-back	1256.06 ± 2900.47^‡^	392.40 ± 458.00	0.7802	δ = −0.0889
SD1	1-back	37.94 ± 43.50	24.37 ± 16.19	0.9682	δ = −0.0222
	2-back	33.38 ± 35.45	20.02 ± 10.25	0.8421	δ = 0.0667
	3-back	31.20 ± 33.93	19.77 ± 12.08^‡^	0.9048	δ = 0.0444
SD2	1-back	51.40 ± 31.67	53.37 ± 23.32	0.3154	δ = −0.2889
	2-back	50.74 ± 28.47	47.57 ± 25.57	0.8421	δ = 0.0667
	3-back	44.86 ± 29.41	44.14 ± 27.84^‡^	1.0000	δ = 0.0000

MON = monolingual; MUL = multilingual; N = number of individuals; † = significant difference between 1-back vs. 2-back within the same group; ‡ = 1-back vs. 3-back; § = 2-back vs. 3-back. Significant within-group differences at p < 0.05. HRV = heart rate variability; SDNN = standard deviation of normal to normal intervals; HR = heart rate; RMSSD= square root of the mean squared differences between successive RR intervals; TINN = triangular interpolation of NN intervals; LF= low-frequency band; HF= high-frequency band; SD1= standard deviation of the Poincaré plot perpendicular to the line of identity (short-term HRV); SD2= standard deviation of the Poincaré plot along the line of identity (long-term HRV)

Within-group effect sizes (paired) for significant indices: MON, Mean RR: † g_rm = 0.7716; Mean RR: ‡ g_rm = 0.8842; Mean HR: † g_rm = -0.7186; Mean HR: ‡ g_rm = -0.9219; LF (ms2): ‡ r_rb = 0.6444; HF (ms2): ‡ r_rb = 0.9111. MUL, SDNN: ‡ g_rm = 0.6863; RMSSD: ‡ r_rb = 0.7091; RR triangular index: † g_rm = 1.0061; RR triangular index: ‡ g_rm = 0.9410; TINN: ‡ r_rb = 0.9556; SD1: ‡ r_rb = 0.7091; SD2: ‡ g_rm = 0.6562

### Task performance differences between MON and MUL participants and within-group across cognitive load levels

Performance data reflected these physiological patterns. MUL participants demonstrated a significantly higher percentage of TP during the 2-back condition (76.67 ± 9.72% vs. 91.00 ± 7.38%; *p* = 0.0020; g = −1.5995), as well as a greater percentage of C (89.14 ± 4.74% vs. 94.67 ± 3.13%; *p* = 0.0075; g = −1.3304) compared to MON. No other performance metrics differed significantly at 1- or 3-back levels (*p* ≥ 0.1613), as shown in Table 5.

Within-group trends were consistent with the expected load–performance trade-off. In both groups, TP and C declined, while FP and RT increased as the task progressed from 1-back to 3-back. Specific post hoc differences are indicated in Table 5 using superscripts: † = 1-back vs. 2-back, ‡ = 1-back vs. 3-back, § = 2-back vs. 3-back (*p* < 0.05).

**Table 5:** Performance Metrics Between MON and MUL Participants and Within-Group Across Cognitive Load Levels

Performance metrics of the *N*-back task		Group		*p*-value (between groups)	Effect size (between groups)
		MON *N* = 9 (M ± SD)	MUL *N* = 10 (M ± SD)		
TP (%)	1-back	98.52 ± 1.76	97.67 ± 3.53	0.8400	δ = 0.0667
	2-back	76.67 ± 9.72^†^	91.00 ± 7.38	0.0020**	g = −1.5995
	3-back	57.04 ± 14.38^‡^	66.67 ± 16.10^‡,§^	0.1890	g = −0.6006
FP (%)	1-back	0.74 ± 1.21	1.17 ± 1.37	0.6299	δ = −0.1778
	2-back	4.63 ± 2.98^†^	3.50 ± 2.54	0.4489	δ = 0.2111
	3-back	10.19 ± 3.27^‡,§^	8.83 ± 3.77^‡,§^	0.4183	g = 0.3641
C (%)	1-back	99.01 ± 0.67	98.44 ± 1.41	0.3537	δ = 0.2333
	2-back	89.14 ± 4.74^†^	94.67 ± 3.13	0.0075**	g = −1.3304
	3-back	78.89 ± 5.96^‡,§^	83.00 ± 6.24^‡,§^	0.1613	g = −0.6427
RT (s)	1-back	0.59 ± 0.07	0.57 ± 0.07	0.4729	g = 0.3222
	2-back	0.82 ± 0.16^†^	0.79 ± 0.16^†^	0.7433	g = 0.1461
	3-back	0.79 ± 0.15^‡^	0.86 ± 0.22^‡^	0.4658	g = −0.3275

MON = monolingual; MUL = multilingual; N = number of individuals; † = 1-back vs 2-back; ‡ = 1-back vs 3-back; § = 2-back vs 3-back. Significant within-group differences at p < 0.05 within the same group. TP = true positives; FP = false positives; C = correct responses; RT = response times. ** indicates a significant between-group difference at that load (p < 0.01). Within-group effect sizes (paired) for significant indices: MON, TP: † r_rb = 1.0000; TP: ‡ r_rb = 1.0000; FP: † r_rb = − 1.0000; FP: ‡ r_rb = − 1.0000; FP: § r_rb = − 1.0000; C: † r_rb = 1.0000; C: ‡ r_rb = 1.0000; C: § r_rb = 1.0000; RT: † r_rb = − 1.0000; RT: ‡ r_rb = − 0.8222. MUL, TP: ‡ r_rb = 1.0000; TP: § r_rb = 1.0000; FP: ‡ r_rb = − 1.0000; FP: § r_rb = − 1.0000; C: ‡ r_rb = 1.0000; C: § r_rb = 1.0000; RT: † r_rb = − 1.0000; RT: ‡ r_rb = − 1.0000

## Discussion

This exploratory study suggests that MUL young adults may combine lower Trait-A and a trend toward lower State-A with subtle but meaningful advantages in WM performance, as well as comparable autonomic flexibility at the group level when cognitive load increases. As that load rose, each group showed load-related changes in autonomic response patterns. Specifically, MUL participants outperformed MON, achieving higher TP rates and overall accuracy at the intermediate 2-back level, despite similar RT and HRV profiles across loads. Taken together, these findings suggest that speaking more than one language may be associated with a specific facet of WM and with reduced anxiety-related interference during cognitively demanding tasks, although this interpretation should be considered preliminary because of the exploratory nature of the study.

Performance on the BANFE-3 was broadly similar across groups, with no statistically significant differences observed across subtests, although a trend toward higher scores in the MUL group was observed for Alphabetical Ordering of Words. In contrast, performance differences emerged only at the 2-back level of the N-back task. Given that the BANFE-3 assesses multiple components of WM and no between-group differences were detected, whereas a difference emerged only at the 2-back level of the N-back task, this discrepancy may reflect task-specific sensitivity to cognitive load rather than generalized WM differences (Calvo et al. 2016). Such effects have typically been reported under conditions of elevated attentional demand (Morales et al. 2013; Comishen and Bialystok 2021), as in the N-back paradigm.

Specifically, at the 2-back level of the N-back task, the MUL group showed higher percentages of TP and C, consistent with previous reports of bilingual advantages at this load (Janus and Bialystok 2018; Barker and Bialystok 2019). Although RT did not differ statistically, MUL participants were numerically faster on 1- and 2-back tasks, indicating that superior accuracy did not incur a speed cost. As expected, the increasing load reduced TP and C while raising FP and RT in both cohorts (Morrison et al. 2019; Comishen and Bialystok 2021). However, the MUL group maintained relatively higher levels of accuracy as task demands increased, particularly at the 2-back level, describing a pattern of more stable performance under higher cognitive demand, and suggesting better attention control when updating longer sequences, although between-group differences in FP were not statistically significant (Janus and Bialystok 2018; Barker and Bialystok 2019; Comishen and Bialystok 2021).

The intersection of anxiety and multilingualism remains understudied in executive-function research. Only Ouzia et al. (2019) have formally examined Trait-A in bilinguals, suggesting a disadvantage in managing adverse emotions. Subsequent work, however, shows that proficient bilinguals can handle emotional distraction more effectively, sustaining superior WM performance (Barker and Bialystok 2019). Previous work has also reported faster target detection under emotional interference (Tao et al. 2023), and bilingual children outperform MON in WM even when emotional regulation is comparable (Janus and Bialystok 2018). High anxiety—State-A or Trait-A—predictably harms WM by competing with processes associated with the storage and attentional resources that should be allocated to tasks (Eysenck et al. 2007; Eysenck and Derakshan 2011; Qi et al. 2014; Moran 2016; Lukasik et al. 2019). In our data, MUL exhibited moderate Trait-A and performed better on 2-back accuracy than MON, whose Trait-A was high. This pattern is consistent with previous evidence linking lower anxiety to better WM performance and may suggest that multilingual experience may be associated with reduced vulnerability to anxiety-related disruptions.

Because anxiety affects selective attention and the ability to ignore distractors (Eysenck et al. 2007), one possible interpretation is that multilingual experience, with its trained attentional system through continuous selection of the target language (Bialystok 2017), may be related to the strengthening of these cognitive processes, even in the presence of Trait-A, which could contribute to the superior WM performance observed in MUL.

At the autonomic level, no group differences emerged, yet load-dependent changes were evident. HRV declined as task demands rose, consistent with earlier findings (Hansen et al. 2003; Byrd et al. 2014; Luque-Casado et al. 2016), which may suggest greater sympathetic engagement or vagal withdrawal to complete tasks efficiently (Knight et al. 2020). In tasks that require greater attentional resources, as well as higher mental workload, mean HR typically rises, accompanied by falls in HF and LF power (Hansen et al. 2003; Chang and Huang 2012; Byrd et al. 2014). Our MON data followed this profile. Mean HR indexes the sympatho-vagal balance: higher values point to sympathetic dominance and reduced HRV, whereas lower values imply parasympathetic influence (McCraty and Shaffer 2015). Both sympathetic and parasympathetic activity contribute to LF, while HF reflects parasympathetic drive; decreases in either frequency band can signal sympathetic activation or vagal withdrawal (Wang et al. 2005; Moses et al. 2007; Delliaux et al. 2019).

Mukherjee et al. (2011) showed that HRV is sensitive to mental effort, with indices such as SD1 and SD2 tracking WM load. They reported decreases in SDNN, RMSSD, and SD2 from easy to complex trials—paralleling our MUL results. Declines in SDNN and RMSSD denote reduced HRV and parasympathetic withdrawal (Shaffer et al. 2014). TINN and the RR-triangular index are less studied: Budhi et al. (2022) linked larger TINN to greater flexibility and better N-back performance, whereas lower RR-triangular values indicate stress and sympatho-vagal imbalance (Hämmerle et al. 2020). SD1 overlaps mathematically with RMSSD (Shaffer and Ginsberg 2017) and thus reflects vagal activity (Mourot et al. 2004). SD2 interpretation is mixed: it may index combined sympathetic–parasympathetic regulation (Delliaux et al. 2019) or drop under heightened State-A (Dimitriev et al. 2016).

Overall, both groups showed a load-dependent reduction in HRV that is consistent with predictions derived from the NIM (Thayer and Lane 2000, 2009). At a theoretical level, this pattern suggests that MUL participants may have engaged regulatory processes shaped by sustained language management to maintain attention and emotional self-regulation by directing resources toward task goals (Bialystok 2017).

HRV indices serve as a common substrate for emotional, cognitive, and physiological regulation via the central autonomic network, anchored in the prefrontal cortex (Thayer and Lane 2000, 2009). The dorsolateral prefrontal cortex, crucial to WM (Cavada and Goldman-Rakic 1989; Brunoni and Vanderhasselt 2014; Vijayraghavan and Everling 2021) also modulates HRV and emotional arousal (Lane et al. 2009). The anterior cingulate, likewise part of this network, is implicated in WM, attention, and anxiety disorders (Lenartowicz and McIntosh 2005; Simmons et al. 2008; Bissonette et al. 2013; Hein et al. 2023). Although the present study did not include direct neural measures, the observed pattern of HRV modulation and task performance in MUL is compatible with these theoretical frameworks, suggesting that multilingual experience may be associated with functional differences in cognitive–autonomic coordination, even under moderate levels of Trait-A.

The present findings suggest that the observed performance advantage in MUL may not be explained solely by lower anxiety levels and may also reflect group differences related to multilingual experience. In contrast, MON exhibited higher levels of Trait-A alongside lower WM performance. Nevertheless, MON still displayed autonomic adaptation, evidencing intact, though comparatively differentiated, regulatory patterns.

## Limitations

Several limitations should be considered when interpreting these findings. The relatively small sample size limited statistical power and the ability to detect potentially meaningful effects. In addition, participants in the MON group had prior exposure to a second language, which may have attenuated between-group differences. Furthermore, HRV recordings were ultra-short (< 5 min) and lacked a resting baseline, which limited detailed inferences about autonomic function.

It is also important to recognize that unmeasured physiological factors—such as individual differences in baseline autonomic tone, respiratory patterns, sleep quality, and physical fitness—might have influenced the variability in HRV responses. Likewise, although the BANFE-3 subtests are theoretically linked to executive and prefrontal functioning, they do not allow direct inferences about underlying neural mechanisms. These mechanisms could be further examined using neuroimaging or electrophysiological techniques, such as electroencephalography (EEG), to correlate working memory performance with specific neural markers.

In addition, some individual factors related to the multilingual language experience that could contribute to the observed differences between groups, such as premorbid intelligence and language-switching frequency, were not assessed. Although the age of second language acquisition was recorded in the MUL group, it was not analyzed as a possible moderator of the observed cognitive or physiological responses. Previous research suggests that age of acquisition and second-language proficiency may influence individual differences in cognitive control, with earlier acquisition and greater proficiency being associated with better performance in some cognitive tasks (Luk et al. 2011; Jiao et al. 2019; Xia et al. 2025). In the present study, English was the second language for all MUL participants, with a mean age of acquisition of 6.7 ± 4.47 years. However, the pilot nature of the study and the limited sample size did not allow us to analyze whether age of acquisition contributed to individual differences in cognitive performance or physiological adaptation.

Ultimately, as an exploratory study, post hoc power analyses revealed heterogeneity; several non-significant comparisons showed power below 80% (see Table S1, Table S2 and Table S3 for more information), and null results should therefore be interpreted cautiously.

Future research should involve larger samples of more strictly defined monolingual participants, obtain resting-state HRV baselines, and include direct neural measures to better characterize the associations among anxiety, multilingualism, and WM performance. Future studies should also use standardized measures of multilingual experience, including age of acquisition, proficiency, language-use frequency, and language-switching frequency, to evaluate their potential moderating effects on cognitive and physiological responses to changing task demands.

## Conclusion

The findings of this pilot study suggest that multilingualism may be associated with differences in working-memory performance, trait anxiety, and patterns of autonomic response to increasing cognitive load. This pattern was accompanied by better working-memory performance, as evidenced by higher true-positive rates and improved overall accuracy in the 2-back condition. This performance pattern was observed despite moderate Trait-A in the MUL group. Heart rate variability, an index of cardiac autonomic activity, did not differ between groups across loads; however, within-group analyses showed that monolingual and multilingual participants exhibited changes in cardiac autonomic modulation as cognitive demands increased. These autonomic changes may be associated with attenuated anxiety-related disruptions in executive functioning while fine-tuning the redeployment of physiological resources that sustain working memory performance. In contrast, MON participants showed both higher Trait-A and poorer performance at the 2-back level; however, the present design does not establish a causal relationship between these findings. Future studies with larger, truly monolingual samples and resting-state autonomic baselines are needed to determine whether multilingualism causally promotes a more adaptive autonomic–cognitive profile.

## Supplementary Information

Below is the link to the electronic supplementary material.

Supplementary Material 1

## Data Availability

The datasets generated and analyzed during the current study are not publicly archived but are available from the corresponding authors on reasonable request.
