# Supplementary material for: Impact of multilingualism on cardiac autonomic activity, working memory and trait anxiety: a pilot study
Source: Cogn Process. 2026 Oct 1;27(4):1475–88. doi: 10.1007/s10339-026-01392-y (PMC13714034; doi:10.1007/s10339-026-01392-y)
Supplement: Supplementary file 1 [file 10339_2026_1392_MOESM1_ESM.docx]

**Supplementary Material**

**Table S1**

Post hoc statistical power for between-group comparisons of STAI and BANFE-3 measures

| Variable | SP (%) |
| --- | --- |
| STAI |  |
| Trait-A (points) | 86.4747 |
| State-A (points) | 32.7621 |
| BANFE-3 |  |
| Total Score (points) | 20.7959 |
| Self-directed pointing (points) | 6.2852 |
| Consecutive subtraction/addition (points) | 37.2008 |
| Visuospatial working memory (points) | 8.2842 |
| Alphabetical ordering of words (points) | 45.1362 |

^Note: SP = Statistical Power; STAI = State-Trait Anxiety Inventory; Trait-A = Trait Anxiety; State-A = State Anxiety; BANFE-3 = Neuropsychological Battery of Executive Functions and Frontal Lobes, 3rd edition.^

**Table S2**

Post hoc statistical power for between-group comparisons of HRV indices and N-back task performance

| Variable | N-back level | Post hoc power (%) |
| --- | --- | --- |
| HRV indices |  |  |
| Mean RR | 1-back | 30.3335 |
|  | 2-back | 36.3914 |
|  | 3-back | 23.2794 |
| SDNN | 1-back | 12.9946 |
|  | 2-back | 9.3380 |
|  | 3-back | 6.7710 |
| Mean HR | 1-back | 30.2716 |
|  | 2-back | 35.5064 |
|  | 3-back | 22.1085 |
| RMSSD | 1-back | 5.0752 |
|  | 2-back | 5.6806 |
|  | 3-back | 5.3015 |
| RR triangular index | 1-back | 11.8179 |
|  | 2-back | 9.3304 |
|  | 3-back | 6.2619 |
| TINN | 1-back | 22.6611 |
|  | 2-back | 7.2387 |
|  | 3-back | 6.3962 |
| LF (ms^2) | 1-back | 24.0003 |
|  | 2-back | 5.3015 |
|  | 3-back | 5.2707 |
| HF (ms^2) | 1-back | 6.9113 |
|  | 2-back | 8.8041 |
|  | 3-back | 6.2158 |
| SD1 | 1-back | 5.0752 |
|  | 2-back | 5.6806 |
|  | 3-back | 5.3015 |
| SD2 | 1-back | 18.9651 |
|  | 2-back | 5.6713 |
|  | 3-back | 5.0303 |
| N-Back |  |  |
| TP (%) | 1-back | 5.6901 |
|  | 2-back | 92.9489 |
|  | 3-back | 25.2579 |
| FP (%) | 1-back | 10.0131 |
|  | 2-back | 12.1262 |
|  | 3-back | 12.2740 |
| C (%) | 1-back | 13.7513 |
|  | 2-back | 81.4980 |
|  | 3-back | 28.2127 |
| RT (s) | 1-back | 10.6637 |
|  | 2-back | 6.1401 |
|  | 3-back | 10.8549 |

^Note: SP = Statistical Power; HRV = heart rate variability; SDNN = standard deviation of normal-to-normal intervals; HR = heart rate; RMSSD= square root of the mean squared differences between successive RR intervals; TINN = triangular interpolation of NN intervals; LF= low-frequency band; HF= high-frequency band; SD1= standard deviation of the Poincaré plot perpendicular to the line of identity (short-term HRV); SD2= standard deviation of the Poincaré plot along the line of identity (long-term HRV);^ ^TP = true positives; FP = false positives; C = correct responses; RT = response times.^

**Table S3.**

Post hoc statistical power for within-group comparisons of HRV indices and N-back task performance

| Variable | Comparison | Group | |
| --- | --- | --- | --- |
|  |  | MON  N = 9  Post hoc power (%) | MUL  N = 10  Post hoc power (%) |
| HRV indices |  |  |  |
| Mean RR | 1-back vs 2-back | 60.3778 | 43.7703 |
|  | 1-back vs 3-back | 72.0018 | 17.6174 |
|  | 2-back vs 3-back | 21.2235 | 10.1939 |
| SDNN | 1-back vs 2-back | 7.5823 | 31.9437 |
|  | 1-back vs 3-back | 21.8117 | 55.4820 |
|  | 2-back vs 3-back | 37.8415 | 28.1435 |
| Mean HR | 1-back vs 2-back | 54.4625 | 45.9430 |
|  | 1-back vs 3-back | 75.4818 | 14.8567 |
|  | 2-back vs 3-back | 28.0873 | 8.3590 |
| RMSSD | 1-back vs 2-back | 15.6389 | 43.2158 |
|  | 1-back vs 3-back | 47.0428 | 60.8779 |
|  | 2-back vs 3-back | 15.6389 | 7.3210 |
| RR triangular index | 1-back vs 2-back | 5.5539 | 86.4857 |
|  | 1-back vs 3-back | 9.8562 | 81.7721 |
|  | 2-back vs 3-back | 11.3316 | 6.8586 |
| TINN | 1-back vs 2-back | 5.3269 | 26.8662 |
|  | 1-back vs 3-back | 16.2761 | 85.5138 |
|  | 2-back vs 3-back | 19.9706 | 38.8760 |
| LF (ms^2) | 1-back vs 2-back | 7.5823 | 34.6738 |
|  | 1-back vs 3-back | 65.4346 | 43.2158 |
|  | 2-back vs 3-back | 47.0428 | 5.5747 |
| HF (ms^2) | 1-back vs 2-back | 37.8415 | 20.0956 |
|  | 1-back vs 3-back | 73.6558 | 34.6738 |
|  | 2-back vs 3-back | 15.6389 | 7.3210 |
| SD1 | 1-back vs 2-back | 15.6389 | 43.2158 |
|  | 1-back vs 3-back | 47.0428 | 60.8779 |
|  | 2-back vs 3-back | 15.6389 | 7.3210 |
| SD2 | 1-back vs 2-back | 5.1616 | 24.1268 |
|  | 1-back vs 3-back | 29.4434 | 51.8520 |
|  | 2-back vs 3-back | 47.4232 | 40.3646 |
| N-Back |  |  |  |
| TP (%) | 1-back vs 2-back | 99.9957 | 63.3694 |
|  | 1-back vs 3-back | 100.0000 | 99.9593 |
|  | 2-back vs 3-back | 96.7275 | 99.9985 |
| FP (%) | 1-back vs 2-back | 92.3313 | 74.4647 |
|  | 1-back vs 3-back | 99.9998 | 99.9957 |
|  | 2-back vs 3-back | 90.9748 | 99.9922 |
| C (%) | 1-back vs 2-back | 99.9974 | 83.9185 |
|  | 1-back vs 3-back | 100.0000 | 99.9994 |
|  | 2-back vs 3-back | 98.9024 | 100.0000 |
| RT (s) | 1-back vs 2-back | 95.9953 | 99.6327 |
|  | 1-back vs 3-back | 80.1974 | 93.2042 |
|  | 2-back vs 3-back | 6.6261 | 18.5969 |

^Note: MON = monolingual; MUL = multilingual; N = number of individuals; SP = Statistical Power; HRV = heart rate variability; SDNN = standard deviation of normal-to-normal intervals; HR = heart rate; RMSSD= square root of the mean squared differences between successive RR intervals; TINN = triangular interpolation of NN intervals; LF= low-frequency band; HF= high-frequency band; SD1= standard deviation of the Poincaré plot perpendicular to the line of identity (short-term HRV); SD2= standard deviation of the Poincaré plot along the line of identity (long-term HRV);^ ^TP = true positives; FP = false positives; C = correct responses; RT = response times.^
